# Shame and HIV: Strategies for addressing the negative impact shame has on public health and diagnosis and treatment of HIV

**DOI:** 10.1111/bioe.12378

**Published:** 2017-08-23

**Authors:** Phil Hutchinson, Rageshri Dhairyawan

**Keywords:** AIDS, HIV, philosophy of emotion, public health, sexual health, shame, stigma

## Abstract

There are five ways in which shame might negatively impact upon our attempts to combat and treat HIV.

1. Shame can prevent an individual from disclosing all the relevant facts about their sexual history to the clinician.
2. Shame can be a motivational factor in people living with HIV not engaging with or being retained in care.
3. Shame can prevent individuals from presenting at clinics for STI and HIV testing.
4. Shame can prevent an individual from disclosing their HIV (or STI) status to new sexual partners.
5. Shame can serve to psychologically imprison people, it makes the task of living with HIV a far more negative experience than it should, or needs to, be.

Drawing on recent philosophical work on shame, and more broadly on work in the philosophy and psychology of emotion, we (a.) propose a framework for understanding how shame operates upon those who experience the emotion, (b.) propose a strategy for combatting the negative role shame plays in the fight against HIV, and (c) suggest further study so as to identify the tactics that might be employed in pursuing the strategy here proposed.

## 1 INTRODUCTION

In this article we propose five ways in which shame plays a negative practical role in public health and in the clinical diagnosis and treatment of HIV. We progress to suggest that a better understanding of shame will enable the development of strategies, which might help people living with HIV to overcome shame and help clinicians to mitigate the effects of shame. We propose that this will contribute significantly to enhancing diagnosis, militating against new infections and improving early diagnosis rates.

There are five ways in which shame negatively impacts on attempts to combat and treat HIV, which emerge from the stigma HIV carries and STI‐stigma in general.

1. Shame can prevent an individual from disclosing all the relevant facts about their sexual history to the clinician.
2. Shame can be a motivational factor in people living with HIV not engaging with or being retained in care.
3. Shame can prevent individuals from presenting at clinics for STI and HIV testing.
4. Shame can prevent an individual from disclosing their HIV (or STI) status to new sexual partners.
5. Shame can serve to psychologically imprison people, it makes the task of living with HIV a far more negative experience than it should, or needs to, be.

Shame, therefore, has consequences, both in the clinical setting and for public health. In what follows, we shall discuss the first four items in our list. We shall then provide a framework for understanding the way shame operates and propose strategies for helping people overcome their shame. We conclude the article by proposing further study.

In outlining a number of ways in which shame might operate as a barrier to good care, effective treatment and public health policy, we shall emphasise the importance of understanding the nature of shame. Such an understanding is of central importance if we are to find effective ways of banishing or mitigating the emotion for the purposes of good clinical and public health practice. For the impact of shame should concern us all. HIV poses a challenge to society as a whole: people living with HIV should not feel ashamed and stigmatised and we should not be facing new infections which might have been avoided in the absence of HIV‐stigma and shame.

While stigma has been extensively discussed in the context of HIV,^1^ the relationship between HIV‐stigma and shame, and how the latter poses serious problems for us, is much less widely discussed. Take, for example, the widely‐read and influential 2003 paper on HIV stigma by Parker and Aggleton^2^ from the journal *Social Science and Medicine*; in this paper, the authors make some important points about conceptual clarity in addressing the problem of stigma, but they do so without discussing shame, as the emotional response to stigma. We would argue that this is akin to proposing strategies and methods for studying the social phenomenon of “threats” while not undertaking, nor even talking about, the fear response to threats. Just as threats are of interest to us as social phenomena because of the impact they have on our lives through the responses they engender, so stigma is of interest to us because of the responses it engenders, the way it makes us feel and behave.^3^ We therefore need to understand those emotional responses, how they are operative, under what conditions and so on.

Moreover, while shame has not gone completely without discussion in the context of HIV, and sexual health more broadly, much of the discussion is undertaken without going below the philosophical ‘waterline’, as it were. Again, Parker and Aggelton's^4^ discussion of stigma is a case in point; for while the task they set themselves is one of arguing for a better conceptual understanding of stigma in HIV research, in undertaking this task they stop short of engaging in the sort of philosophical work in the epistemology and ontology of stigma that is, we suggest, required for better understanding. Instead, they proceed to select from among a number of social theories, their favourite being a reworking of Goffman, to incorporate insights from Foucault and Bourdieu. The point we want to emphasise is this: it is crucial to be well‐versed in the philosophical discussions about human emotions, and shame, and have these discussions inform one's practice, if that practice is to be effective. Misidentifying the nature of shame and stigma will lead to ‘misfiring’ attempts to address shame and stigma.

One of the central claims for which we are arguing here is that theories of shame and stigma are not what is required (either philosophical theories or social theories). As we will argue below, shame is a response to the meaningful lifeworld, and the meaning the lifeworld has for an individual draws on the individual's enculturation, their interests, and the socio‐political context. Because shame draws on resources that exist at high levels of cultural specificity, there are likely to be significant differences in the specifics of shame and stigma across cultures, socio‐political contexts and relative to the specific individual involved. We therefore want here, in this article, to argue for a framework which affords us a sensitivity to such cultural, socio‐political and individual specifics that comprise actual shame and stigma experiences in specific contexts.

The bio‐chemical^5^ advances in the treatment of HIV rank alongside the foremost achievements of modern biomedicine, and perhaps even beyond. In contrast, while the psycho‐social aspects of HIV are widely acknowledged as being constitutive of the distinctive pathology of the virus, the extent to which we have understood the nature of, and developed effective treatments for, HIV stigma and shame are marked by a distinct lack of progress. We hope that this article will go some way to reducing the inbalance between bio‐chemical progress and psycho‐social inertia.

## 2 UNDERSTANDING SHAME

When shame is discussed in the philosophical and psychological literature, it is usually depicted as a (higher) cognitive or complex emotion of self‐assessment.^6^ While there are some problems inherent to this depiction, it serves as an appropriate starting point. When one unpacks this categorisation, it means simply that the experience of shame, its felt quality, or what we might call its *phenomenology,* is characteristically constituted and type‐individuated by evaluative beliefs about the self (or a situation of which the self is a constituent part). Where guilt is primarily associated with a belief that one has *acted* in a transgressive manner, violating a law or social norm, shame, it is often proposed, operates on one's being: it is less about what one does and more about who one is, and how this might stand in relation to a person's awareness of how others perceive them. In addition, shame often leads to a desire to hide, to withdraw, from the (life‐)world.

Although this will stand as a preliminary depiction, it is important not to be misled. One of us, Hutchinson,^7^ has proposed that the evaluation which gives rise to shame operates not at the level of propositionally‐structured evaluative beliefs or judgements, but rather at the level of conceptual frames.^8^ It will become clear why this is significant as we progress.

### 2.1 Autonomy and heteronomy

To feel shame is to regard oneself as worthy of shame, and this shame‐worthiness can have a number of sources. It can be *heteronomous*, so that shame is an acknowledgement, a taking‐on‐board, of the judgements (or morally‐loaded perceptions) of others about one's self, and in so doing considering oneself, one's being, to be in some way evaluatively diminished. Shame can also be *autonomous*, so that shame serves as testament to a mismatch between the sense of self one assumes and seeks to project to others and the self that one considers oneself to be on reflection.

Autonomous shame, therefore, might emerge from a mismatch prompted by acknowledging *all* one's actions and all one's beliefs, rather than focusing only on some convenient or perhaps self‐serving selection of these. Take an example: consider the “liberal” who must acknowledge their sexism or racism when they have reflected on their subtle‐but‐there‐all‐the‐same propensity to racial or gender stereotype. In this case, shame might emerge as our “liberal” comes to acknowledge the tension between this aspect of their character – their propensity to subtle gender‐ or racial stereotyping – and the liberal character they had assumed and projected as theirs. Shame, the emotion, can testify to this (if they are merely embarrassed they really haven't acknowledged the true significance of the tension). This provides us with an example of autonomous shame.

However, as we have already remarked, it is a characteristic of the emotion that one can experience shame owing to tensions that testify to mismatches between social norms or mores on the one hand and aspects of one's character on the other. Here the self‐evaluation that one falls short of some standard relates to a standard which transcends and exists external to the character of the specific individual. This is the kind of shame which testifies to perverse social norms and mores, the sort of shame in evidence when one considers the shame that some rape victims experience. Here the tension has its source in the way in which social norms intersect with one's sense of self and how this, despite one's beliefs about oneself, seems to impose upon one a sense of shame. This type of shame is often accompanied by a desire to flee or hide from others, from the society (the audience, the honour group) that has conferred upon one this shame. This provides us with an example of heteronomous shame.

While there has been much discussion of whether shame is a characteristically heteronomous or autonomous emotion,^9^ Hutchinson^10^ has argued that it can appear to operate, from case to case, either autonomously or heteronomously, but that ultimately, when one looks closely enough at the sources of shame, the distinction collapses. This is an important point on which to be clear, because if one holds that shame is always autonomous, that might well lead one to focus any attempt to alleviate shame solely on the psychology of the individuals who bear shame. One will see shame as a purely psychological problem for individuals. Conversely, if one were to assume that shame is always heteronomous, that shame is instantiated in individuals by their acceptance of the judgement of others who form their honour group, then that will lead one to identify that which is in need of change as being the social norms which the honour group (the shame‐instantiating audience) embody (and/or the attachment the individual experiencing shame has to those social norms). In this latter, heteronomous sense, therefore, addressing shame might be a political, cultural and social task in addition to being a psychological task.

To provide an example, the rape victim who feels shame for the tainting of the family name that has resulted from their rape might well benefit from psychological treatment, which facilitates their detachment from the social norms that stigmatise rape victims and their families. However, our proposal is that it would be crass to believe that this is all that needs addressing in response to the shame experienced in such cases. The social norms and the honour groups that embody them need subjecting to criticism and transformation. The project must be both psychological *and* political.

### 2.2 Shame: Causal stimuli and response, belief and expectation or meaning relations?

Shame does not operate in a manner that can be captured through causal explanation of how a person relates to their world, such as in terms of stimulus and response mechanisms. Neither is it apt for understanding in terms of a person's beliefs or judgements about their world and others sharing that world. Shame can simply descend on one; one can be struck by shame. Shame operates at the level of meaning relations, where a person takes in their meaningful – their conceptually‐saturated – world. The world understood as the world as we experience it, as conceptually available to us, is what we mean to invoke in employing the term *lifeworld*. Understanding shame, therefore, requires a way of re‐presenting these meaning relations in all their richness, and not seeking to reduce them to cognitivist (propositional beliefs and Judgements) or Jamesian (causal) accounts. Hutchinson^11^ proposed a framework for making sense of shame expressions, where they might pose difficulty; this framework was labelled “world‐taking cognitivism”, and while the label is ultimately unimportant, unpacking it serves to bring out the reasons for proposing the framework.

- The “world‐taking” part is there to emphasise that when we seek to make sense of a particular emotional expression, that with which we need concern ourselves is the way in which the person expressing the emotion has taken‐in the lifeworld: how they have “read” the world, or the situation, how they have conceived it. This is a meaning relation, as opposed to a causal one, such as stimulus and response.
- The “cognitivism” part is employed in the way that term is used in analytic meta‐ethics (not in cognitive psychology or cognitive science) and merely invokes a commitment to the idea that the thoughts in which we are here interested are responsive to the world.

So, “world‐taking cognitivism” proposes that one makes sense of emotional expressions as based in a person's takings of loci of significance in a meaningful world (lifeworld), to which those emotions are answerable. Understanding an emotional expression will therefore be arrived at through reconstructing the (internal) relationship that holds between a person's conceptualisation of a situation (including their conceptualisation of self) and the concept of the emotion. Put simply, shame stems from a person's perceiving, or “taking” a situation involving them as characteristically shameful, and their being, who they take themselves to be, is tainted in and through having taken things in this way.

Shame emerges from meanings encoded in our language at a more fundamental level than is captured by a focus on propositionally‐structured beliefs, what we might call *belief that*. What this means is that shame can often remain untouched by demonstrating to a person who feels shame that their shame does not find support in rationally‐defensible beliefs about the lifeworld and that person's role in that lifeworld. Shame often rests on framing concepts, that is to say, those concepts which frame one's world‐view. Treating shame (often) requires reframing.

This way of understanding shame finds support in recent work on the placebo response, by authors such as Moerman^12^ and Benedetti.^13^ Here also, stimulus‐response and belief‐expectation explanations are shown to be inadequate and meaning relations are proposed to explain the ‘placebo’ response.

## 3 SHAME AND HIV

We remarked at the beginning of this article that shame impacts upon diagnosis and treatment and on public health concerning HIV. In what follows, we explore this further.

### 3.1 Shame's impact on diagnosis and treatment

Good treatment relies on accurate diagnosis, but it also relies on the patient following the designated treatment regimen once they've been diagnosed. These are two aspects of the clinical context in which shame and stigma have an impact.

#### 3.1.1 The clinical encounter and diagnosis

A significant hindrance to any attempt at diagnosis and therefore, by extension, good treatment can be located in a patient's willingness and ability to fully and honestly disclose relevant details in response to the questions of health practitioners. Knowledge is central to diagnosis, and something acting as an impediment to that knowledge in turn acts as an impediment to accurate diagnosis and to good treatment. Of course, the relevance and significance of the role of patient disclosure can vary across health contexts. Our suggestion is that in the area of STI diagnosis and treatment, particularly with regards to HIV, the significance is high. It is important, perhaps crucial, for the health practitioner to have available to them all the relevant facts, without that availability being diminished by the patient withholding information. Unfortunately, HIV treatment is also the context in which shame is often operative as a prominent source of a patient's resistance and failure to disclose. This is because of the stigma‐effect. Illness and infections which carry social stigma give rise to shame and that shame can serve as a hindrance to full disclosure of the relevant facts. We will illustrate this point with our first case study (CS1).

### 3.2 Case study one (CS1 – shame and disclosure)

Susan, a 35 year old woman, attends A&E presenting with worsening headaches. She is admitted under the neurology team and undergoes several investigations which are reported as normal. Four days into her admission, her headaches worsen and she undergoes an MRI scan, which shows generalised cerebral oedema (brain swelling). Since HIV can be one of several causes for this, the medical team suggest an HIV test, and she reveals that she was diagnosed with HIV several years earlier at another hospital. She was being looked after by the HIV service at that hospital and was doing well on antiretroviral medication. She told them that she had decided not to tell them this information as she did not feel it was relevant to her current problem. She also reported that she had suffered considerable stigmatising behaviour from the non‐HIV specialist health care professionals at the other hospital, which put her off telling anyone unless it was necessary. She had continued to take her antiretroviral medication on the ward secretly. With this new information, the neurology team contacted the local HIV specialist unit who arranged for her to be transferred to them the next day. Unfortunately, before this could happen, Susan collapsed on the ward and died. The cause of death was thought to be the HIV‐related brain disease.

It is reasonable to conclude, that had Susan felt able to disclose her full medical history to her medical team, they would have been able to access specialist HIV advice sooner, which might have saved her life. The shame she experienced following her diagnosis affected her ability to disclose her HIV status.

#### 3.2.1 Long term treatment and retention in care

Currently there is no known cure for HIV. Successful treatment of HIV involves taking antiretroviral medication daily for the rest of an individual's life (adherence), and attending HIV services regularly (retention in care). This is known to be challenging in many chronic diseases such as diabetes and hypertension, where good adherence is thought to be > 60% of drug doses taken correctly. It is even more challenging in HIV, where in order to avoid drug resistance >95% of drug doses need to be taken correctly.

Episodic treatment interruptions cause problems. First, there is some evidence to suggest that they increase drug resistance, where a person whose ART regimen is interrupted responds less well to their ARVs as a result. Second, breaks from treatment increase the chance of a patient's health deteriorating, their developing clinical AIDS or even of their death. The SMART trial, which was a large International study involving just short of 5500 volunteers in 33 countries comparing continuous with episodic treatment of HIV, was stopped early when the evidence became clear that the patients who were following episodic treatment were at twice the risk of developing clinical AIDS.^14^

Given the nature of stigma associated with HIV, and the shame that emerges from this, it is reasonable to expect that shame and stigma might be contributory factors in a person's decision to stop or take a break from treatment, and it is this with which we are concerned here. While, from a narrowly‐conceived bio‐medical perspective, the ART might well enable an individual to live a normal life, from a psycho‐social perspective that same therapy – taking the pills, attending the clinic, the blood tests – might serve to continually remind the person of their HIV status and the stigma associated with HIV. The very thing that bio‐chemically supresses the virus can serve, psycho‐socially, to trigger stigma and shame. We illustrate this with our second case study (CS2).

### 3.3 Case study 2 (CS2 – shame and non‐engagement in care)

Jamie, a 47‐year‐old man, was diagnosed with HIV during a routine sexual health test. He was referred to the HIV service within the same hospital but did not attend his appointment for assessment. The HIV clinic contacted him regularly by email to encourage him to come in and he responded, but said that he did not feel able to come in and he was dealing with the diagnosis himself. One year later, he developed bloody diarrhoea, opening his bowels up to ten times a day. He was admitted to hospital and disclosed his HIV status to the medical team. Investigations showed that he had advanced HIV infection with a very low CD4 count and cytomegalovirus colitis, an opportunistic infection found in very immunosuppressed individuals. He was started on treatment but then developed pneumocystis jirovecii pneumonia requiring intensive care support. He died 2 months later from the complications of advanced HIV and several opportunistic infections.

Had Jamie felt able to attend the HIV clinic at diagnosis, he would have been recommended to commence antiretroviral treatment earlier and may not have developed life‐threatening illnesses due to immunosuppression.

### 3.4 Shame and public health

Combatting HIV does not begin and end with diagnosis and treatment, it also has a large public health component, which is not reducible to vaccination. Vaccines simply don't exist for many STIs, and an HIV effective vaccine is not imminent. Nor is prophylaxis a public health *solution*. For example, in the case of HIV, post‐exposure prophylaxis (PEP) is expensive, is associated with quite significant side effects for some patients, involves strict regimens, and a small time‐window between exposure and effective administration. Pre‐exposure prophylaxis (PrEP) for HIV is more viable, and we believe should play a part in the fight against new infections. However, even here there are significant problems, in addition to some of those associated with PEP, such as possibly contributing to drug resistance. However, perhaps the problem most relevant to our discussion here is the current debate about PrEP as a sanctioned public health intervention. Here, those who argue against the use of PrEP often argue against it on the grounds that it encourages ‘unsafe’, ‘risky’ sexual practices, or sexual practices that shouldn't be encouraged from the perspective of specific moral viewpoints. Such arguments might serve to stigmatise those who want PrEP. We will leave this observation here, and focus instead on disclosure and testing.

So, the public health component of the battle with STIs has multiple components, of which two are: disclosure to new sexual partners and presentation for testing.

#### 3.4.1 Shame and disclosure to new sexual partners

HIV‐stigma can give rise to a sense of shame, and one of the ways in which individuals might seek to manage this is through withholding their HIV status. For when one knows others are unaware of one's status then their gaze cannot be shaming. Moreover, if shame and stigma serve to prevent an individual from disclosing their status to a sexual partner, then it is, perhaps, reasonable to assume that there might be, for some, a fear of requesting condom use, based on the same shame and stigma: for if disclosing one's HIV status is to risk providing evidence to a potentially ingnorant and prejudiced sexual partner, then suggesting condom use can be seen as a risk by providing clues or hints of one's status to that same prejudiced sexual partner. While from this perspective withholding one's status can seem like a rational response to the fear of shame, it might have rather catastrophic consequences from the perspective of public health. For, not only is it likely to contribute to increased infections but it is also likely to contribute to the problem of late diagnosis, because a person who has no reason to believe they have been exposed to infection has little reason to the believe they should be tested for infection. In the case of infections with prolonged incubation periods, such as HIV, this is particularly relevant. The decision to disclose or not *is* a difficult one. Disclosing to the wrong person can lead to prejudice, stigma, ostracisation and even intimate partner violence. We do not propose that combatting HIV‐stigma and shame is desirable because it will enable us to achieve full disclosure. A person's decision whether to disclose or not can be based on many factors, we are only conerened that stigma and shame should combatted as prominent among these factors, where possible. The desire for privacy should always be respected. It is not clear to us, for example, that an individual with a fully suppressed viral load has any moral obligation to disclose their status.

#### 3.4.2 Shame and testing

The act of attending a clinic for a test can be shaming in itself, in that the person perceives their attendance at the clinic for testing serves as a confirmation or admittance that they warrant the suspicion that they might be infected. In this sense the act of attending a clinic for testing can serve to reinforce an already‐existing framing of STIs and HIV as shameful. The act of attending clinic and presenting oneself for testing can serve to validate or confirm the underlying framing‐concepts. If STIs are stigmatised, then attending an STI clinic and taking a test for an STI serves to reinforce the shame that might be felt in response to that stigma. It is difficult to overstate the problem this presents for public health programmes. Regular testing is, from a bio‐medical perspective, crucial, yet the very act of testing seems to exacerbate the psycho‐social problem posed by STI‐ and HIV‐shame.

That being said, there is evidence that many people diagnosed late *have* been in touch with health care professionals in the recent past, yet have not been offered tests. It has been shown that barriers to testing are often equally, if not more, likely to be put in place by the clinician than by the patient. Such barriers might include poor knowledge about how HIV may present and, among non‐sexual health clinicians, whether a test is needed; it might also be the case that the health care professional doesn't know that there is no longer a requirement for lengthy pre‐test counseling. In addition, reticence in offering an HIV test might: a) emerge from the healthcare professional's own embarrassment and shame in talking about HIV and sexual health, or b) emerge from a fear of embrassing, shaming or insulting the patient.

Contrast the observations of the previous paragraph with what we know about settings where HIV testing is routine and “opt‐out” testing rates are high. For example, in antenatal clinics where all women are offered tests by midwives, the uptake rate is 98%^15^ (Yin & contributors, 2014). In order to decrease late diagnosis rates and encourage HIV testing in non‐specialist sexual health/genitourinary medicine settings, national HIV testing guidelines were written in 2008,^16^ which aimed to normalize testing. These suggest settings and clinical situations where testing should be routine, with the hope that by normalizing the test, more clinicians will offer them. This might negate the shame felt and transmitted by the clinician in talking about HIV, which in turn might make it more possible for the patient to accept the test.

### 3.5 Acknowledging the extra‐bio‐chemical aspects of HIV pathology

The problem we continue to face might be stated as follows: the bio‐chemical and the psycho‐social are fully intertwined in the pathology of HIV, and to make this claim is uncontroversial. We know that poverty, culturally‐bestowed attitudes to sex and sexuality, laws on sex work, drug use, immigration, and poor mental health, to name but a few items from a long list, are significant drivers of infection rates, take‐up of testing, and development of clinical AIDS. Believing we can achieve good clinical treatment and public health policy without taking full account of the psycho‐social aspects of HIV pathology is folly. We submit that such folly ranks alongside that of someone who assumed we might achieve good treatment through ignoring bio‐chemical treatment of the virus through ARVs while merely focussing attention on the psycho‐social aspects. While the methods of social inquiry and those of biochemistry differ, and do so in significant ways, what is common to both is the importance of understanding the nature or character of the phenomena;^17^ a first step in addressing shame and stigma should be to gain understanding of the nature of shame and stigma in the context of HIV.

## 4 ADDRESSING SHAME IN THE CONTEXT OF HIV

Shame and stigma operate on individuals at a deep psychological level, which can make them difficult to overcome. Put another way, shame can often be experienced by an individual who concurrently believes that they have nothing about which to feel ashamed. (This is often depicted as one of the defining characteristics of shame). So, pointing out to someone that there is no reason to feel ashamed of their HIV status or their sexual behaviour when responding to the confidential questions posed by a clinician at a STI clinic, will often leave untouched the shame which that individual feels. This is because our emotional reactions are based on the meaning the social world (lifeworld) has for us and the way that is mediated through our language. Invariably, the ways such meaning is mediated does not operate at the level of beliefs one has about the world, but might rather stem from the way certain meanings are metaphorically encoded in our language, and therefore structure or frame our beliefs.

It is a characteristic of much shame experience that it is akin to a *feeling* of guilt, which occurs accompanied by a clear sense that one is *not* guilty of anything. Providing someone who has been raped and feels shame with a set of reasons for why they are guilty of nothing will often leave their shame untouched, because the shame functions at a more fundamental level than does guilt. Shame is rarely constituted by a set of evaluative beliefs, but is rather based in the way our language, at a pre‐propositional level, frames our reading of the lifeworld: our conceiving of the world. This understanding shows us that relieving shame is not about refuting false beliefs or replacing them with true ones, but a matter of facilitating the decoding of the meanings which are conferred, often by stealth, by the linguistic frames.

One illustration of this point is to look at the ways people sometimes communicate about aspects of sexual health, such as a negative HIV test result being communicated as “I'm clean”, thereby implying that infections are “dirty”.^18^ While a person might well believe (and rightly so) that there is nothing “dirty” about having contracted, or carrying, an infection, the fact that such metaphors are operative, are encoded in the way we talk about STIs, means that at a deep psychological level, irrespective of what one might believe, sexually‐transmitted infections are thereby framed in terms of cleanliness and dirtiness. Here the conceptual metaphor^19^ of cleanliness/dirtiness, and the moral connotations these concepts carry with them, lead to a kind of *moral* framing of an otherwise amoral, or morally inert, test result. So, while at the level of propositionally‐structured belief, the recipient of a positive HIV test might very well, rightly, take the test result in a completely morally neutral way, at a deeper level, at the level of framing metaphors, the result is morally‐cast. It is as if the metaphor of cleanliness/dirtiness serves to colour or taint the meaningful content of the test result in a way which diminishes the very being of the person who has had a positive result. The person *feels* themselves to be viceful, while concurrently being clear in their *belief that* they have committed no immoral act. Their being is diminished.^20^

Because shame operates at this level, nullifying it or combating it requires appropriate methods. One cannot seek to combat shame by simply presenting to those afflicted by shame an argument that they have transgressed no rules and therefore their feeling of guilt is unwarranted. To re‐employ the “cleanliness” example, the task is to first bring the person who is ashamed to the realisation that this metaphor of cleanliness‐dirtiness is operative in their subconscious and the moral framing that it operates to create. The method for combating or nullifying shame is one of identifying the source of the shame, how it is encoded in one's way of framing the world. Bringing this encoding to consciousness will in itself do much to break the grip of shame.

## 5 PROPOSALS FOR FUTURE STUDY

What we have set out to do in this article is bring to bear insights from the latest philosophical work on emotions and shame. Our hope is that this can lead to the development of strategies which can be implemented, thereby serving to diminish the obstructive role of shame in the clinical and public health treatment of HIV.

Shame can be addressed at two levels: we can look at the socio‐political drivers of shame, and how these become absorbed into our ways of speaking. Here a stigmatisation which might begin as a socio‐political attitude, even as a government policy or legal prohibition, might get fixed in the conceptual methaphors we employ long after attitudes, policies and laws have changed. Shaming, on this understanding, can be intentional or structural.^21^ While a shaming attitude might very well no longer be current, or prevalent, that does not mean that the shaming effects have passed into history; those shaming effects might still be present in our modes of expression. So, in addition to addressing ourselves to current socio‐political attitudes, which feed HIV‐stigma, like those documented in Norman Fowler's recent book *AIDS: Don't Die of Prejudice,* 22 we also need to analyse our language use, including in public health messaging, so that we might ensure that we are not reinforcing‐by‐stealth HIV‐stigma and shame.^23^

We propose that studies be undertaken which analyse the conceptual metaphors employed in public health literature and the effects these exercise on individuals. In addition to such a linguistic analysis, we propose deliberative fora (building on Fulford's Values‐Based Practice) wherein stakeholders can reflect on their own communication and value assumptions.^24^

Whether intentional or structural, these socio‐political and linguistic sources of shame serve as sources because of the way they are taken on, or acknowledged, by individuals. A person's shame might have its source in conceptual metaphors employed in talk about them and their condition (structural) or in pro‐active depictions of people with that condition by others (intentional). In both cases, in addition to addressing the sources (as proposed in the previous paragraph), a person can be supported and helped in the process of detaching from the evaluation conferred upon them by the construals, judgements or linguistic acts of others or by the structures. In the UK, psychological support for people living with HIV is recommended by BHIVA^25^ and many clinics have dedicated sexual health psychologists. This is crucial to good HIV treatment. While HIV remains stigmatised, those living with the virus deserve psychological support to help them detach from the stigma, thereby mitigating the shame. Further research on the most effective way of accomplishing this would be beneficial.

More than merely a virus, HIV serves as a vector through which flow many of society's already‐present prejudices. Shame often emerges from these prejudiced gazes. One can feel ashamed of one's sexuality, one's sexual behaviour, one's poor health, one's dependency on healthcare, one's immigration status, one's low socio‐economic status, one's failure to heed the warnings in the public health messages, and on and on. HIV serves to catalyse these potential sources of shame into what we observe as HIV‐shame and stigma. Understanding the nature of HIV‐shame and stigma will enable us to mitigate its effects.

## Footnotes

1
See, for example: Altman, D., Aggleton P., Williams M., Kong T., Reddy V., Harrad D., … Parker R. (2012). Men who have sex with men: Stigma and discrimination. *The Lancet, 380*(9839), 439–445; Anderson, M., Elam, G., Gerver, S., Solarin, I., Fenton, K., & Easterbrook, P. (2008). HIV/AIDS‐related stigma and discrimination: Accounts of HIV‐positive Caribbean people in the United Kingdom. *Social Science & Medicine, 67*(5), 790–8; Berger, M. T. (2004). *Workable sisterhood: The political journey of stigmatized women with HIV/AIDS* Princeton and Oxford: Princeton University Press; Bernays, S., Rhodes, T., & Janković‐Terzić, K. (2010). You should be grateful to have medicines: Continued dependence, altering stigma and the HIV treatment experience in Serbia. *AIDS Care, 22*(suppl. 1), 14–20; Bharat, S., (2011). A systematic review of HIV/AIDS‐related stigma and discrimination in India: Current understanding and future needs. *SAHARA J, Journal of Social Aspects of HIV/AIDS Research Alliance, 8*(3), 38–48; Bravo, P., Edwards, A., Rollnick, S., & Elwyn, G. (2010). Tough decisions faced by people living with HIV: A literature review of psychosocial problems. *AIDS Reviews, 12*, 76–88; Campbell, C., & Deacon, H. (2006). Unravelling the contexts of stigma: From internalisation to resistance to change. *Journal of community & applied social psychology, 16*, 411–417; Campbell, C., Foulis, C. A., Maimane, S., & Sibiya, Z. (2005). I have an evil child at my house: Stigma and the HIV/AIDS management in a South African community. *American Journal of Public Health, 95*(5), 808–815; Campbell, C., Nair, Y., Maimane, S., & Nicholson, J. (2007). Dying twice: A multi‐level model of the roots of AIDS stigma in two South African communities. *Journal of Health Psychology, 12*(3), 403–416; Castro, A., & Farmer, P. (2005). Understanding and addressing AIDS‐related stigma: From anthropological theory to clinical practice in Haiti*. American Journal of Public Health, 95*(1), 53–59; Collins, P. Y., von Unger, H., & Armbrister, A. (2008). Church ladies, good girls, and locas: Stigma and the intersection of gender, ethnicity, mental illness, and sexuality in relation to HIV risk. *Social Science and Medicine, 67*, 389–397; Dodds, C. (2006). HIV‐related stigma in England: Experiences of gay men and heterosexual African migrants living with HIV. *Journal of community & applied social psychology, 16*, 472–480; Green, G. (1995). Attitudes towards people w HIV: Are they as stigmatising as people with HIV perceive them to be? *Social Science & Medicine, 41*(4), 557–568; Haile, R., Padilla, M. B., & Parker, E. A. (2011). Stuck in the quagmire of an HIV ghetto: The meaning of stigma in the lives of older black gay and bisexual men living with HIV in New York City. *Culture, health & sexuality, 13*(4), 429–442; Kalichman, S. C., Simbayi, L. C., Cloete, A., Mthembu, P. P., Mkhonta, R. N., & Ginindza, T. (2009). Measuring AIDS stigmas in people living with HIV/AIDS: The internalized AIDS‐related stigma scale*. AIDS Care, 21*(1), 87–93; Parker, R., & Aggleton, P. (2002). *HIV/AIDS‐related stigma and discrimination: A conceptual framework and an agenda for action*. The Population Council Inc. 10.1016/S0140-6736(12)60920-922819652

2
Parker, R., & Aggleton, P. (2003). HIV and AIDS‐related stigma and discrimination: A conceptual framework and implications for action. *Social Science & Medicine, 57*, 13–24. 10.1016/s0277-9536(02)00304-012753813

3
Investigations which are concerned to focus upon how phenomena such as threats and stigma can be used as tools of social control are also predicated on understanding these phenomena as objects of fear and shame, respectively.

4
Parker, R., & Aggleton, *op. cit.* note 1.

5
We use “bio‐chemical” to denote, specifically, drug interventions. We prefer this to the term “bio‐medical” in this context, because this latter term both should and in fact does incorporate much of the psycho‐social considerations we are concerned with here. A clinician's identification of appropriate treatment of, for example, viral infection is based not *only* on knowledge of the best bio‐chemical intervention, which is read‐off the current RCT data (or meta‐analyses), but also on judgements about the patient, which involve judgements about their ability to adhere to drug regimens, and so on. Therefore, it strikes us as misleading to contrast the bio‐medical with the psycho‐social. Bio‐medicine is, we believe, best understood as a practice which incorporates bio‐chemical expertise with biological (physiological) knowledge, embedded in a background understanding of the ways in which psycho‐social factors penetrate and influence health. We want to bring the background to the fore.

6
Taylor, G. (1985). *Pride, shame and guilt: Emotions of self‐assessment* Oxford: Oxford University Press; Griffiths, P. E. (1998). *What emotions really are: The problem of psychological categories* Chicago: Chicago University Press; Hutchinson, P. (2009). Emotion‐philosophy‐science. In Y. Gustafsson, C. Kronkvist & M. McEachrane (Eds.) *Understanding Emotions*, Basingstoke: Palgrave Macmillan; Hutchinson, P. (2008). *Shame and philosophy: An investigation in the philosophy of emotions and ethics*. Basingstoke: Palgrave MacMillan.

7
Hutchinson, *op. cit.* note 6; Hutchinson, P. (2015). Shame, placebo and world‐taking cognitivism. In S. George & P. Jung (Eds.) *The Cultural Ontology of the Self in Pain* (p. 165–181). Springer.

8
In addition to Hutchinson, *op. cit.* note 6, see also Kovecses, Z. (2000). *Metaphor and emotion*. Cambridge: Cambridge University Press.

9
Williams, B. (1994). *Shame and necessity* Berkley: University of California Press; Dilman, I. (1998). Shame, guilt and remorse. *Philosophical Investigations. 22*(4), 312–329.

10
Hutchinson, *op. cit.* note 6.

11
Ibid; Hutchinson, P. (2011). Facing atrocity: Shame and its absence. In *Passions in Context, 1*(2).

12
Moerman, D. (2002). *Meaning, medicine and the placebo effect*. Cambridge: Cambridge University Press.

13
Benedetti, F. (2014). *Placebo effects* (2nd ed.). Oxford: Oxford University Press.

14
SMART Study Group. (2006). CD4+ count–guided interruption of antiretroviral treatment. The strategies for management of antiretroviral therapy. *New England Journal of Medicine, 355*, 2283–2296. 10.1056/NEJMoa06236017135583

15
Yin, Z., Brown, A. E., Hughes, G., Nardone, A., Gill, O. N., Delpech V. C., & contributors. (2014). *HIV in the United Kingdom 2014 Report: Data to end 2013*. Public Health England. London.

16
UK National Guidelines for HIV Testing (2008). Retrieved from http://www.bhiva.org/documents/guidelines/testing/glineshivtest08.pdf

17
Indeed, it is this – the understanding of the differing nature of the phenomena under investigation in each domain of inquiry – that informs us as to the appropriate methods of inquiry in each domain.

18
Some readers might assume we are here invoking Mary Douglas's work on dirt and pollution. We are not doing so. It is important to our purposes here that what we write is not taken as invoking a substantive theoretical account of dirt and pollution, such as that Douglas advanced. We are simply employing the example of the clean/dirty metaphor to show how an otherwise morally neutral social practice/medical procedure can come to have moral significance at the sub‐propositional level – i.e. without an individual believing that they are worthy of moral sanction – because of the framing metaphors. We could just as easily have employed a different example (“free” instead of “clean”, perhaps), which would have invoked another kind of evaluation, sub‐propositionally. What we want to do here is to draw attention to and give an example of the way in which people can feel ashamed of something, while concurrently believing that they have nothing to be ashamed of. They can do so because evaluative “tainting”, so to speak, can enter via these conceptual (as opposed to literary) metaphors; what are sometimes referred to as “framing metaphors”. To be sure, if one sought to unpack the specific ways in which this particular framing metaphor imports a specific evaluative stance and the way in which this might lead to shame and stigma, then one would certainly want to consult Douglas's work at the outset. But this is not our task here. We are making an argument for *why* framing metaphors should be an important locus of investigation when one seeks to understand shame and stigma. Moreover, that investigation should begin with an examination of conversation, and the metaphors employed in those conversations, as opposed to with theories. See Douglas, M. (1966). [2002]. *Purity and danger: An analysis of concepts of pollution and taboo*. London: Routledge.

19
Conceptual metaphors are what used to be referred to as ‘dead metaphors’; metaphors which are so embedded in our language and ordinary ways of communicating that we are often unaware of their metaphorical status. The conception of conceptual metaphor we here employ can be ultimately traced to the later philosophy of Ludwig Wittgenstein. It was given programmatic treatment by George Lakoff and Mark Johnson, and has since been developed into what is now called cognitive linguistics. We do not subscribe to the programme of cognitive linguistics, but believe there are important insights to be found in Lakoff and Johnson's work on metaphor, taken alongside Wittgenstein's work. See Wittgenstein, L. (1958). *The philosophical investigations* Oxford: Blackwell; Lakoff, G., & Johnson, M. (1989). *Metaphors we live by*. Chicago: Chicago University Press.

20
We don't claim that these framing or metaphor effects are determinate. Some cognitive linguists seem to imply that the effects of framing (conceptual) metaphor are determinate (e.g. Kovecses, *op. cit.* note 8). We propose that this is an open question, to be answered through further study, and that it is not something which can be claimed as an acontextual theoretical truth, as it were. It is for this reason that we resist the depiction of conceptual metaphors as “cognitive metaphors”, as one finds them depicted in the cognitive linguistics literature. We propose that one can gain better understanding of the metaphor effects by engaging in conversational analysis of expressions in which such frames are invoked. What is clear is that such metaphors carry over from their source domains a set of grammatical constraints, which then come to exert control in the target domain. So, the moves one might make with the concept of “clean” in the context of an HIV test are to an extent constrained by the grammar of “clean”, as that grammar is imported from the source domain (where clean is employed non‐metaphorically). However, the effects such conceptual metaphors might have on individuals beyond these inherited grammatical constraints will be a matter for investigation, not theory.

21
An anonymous reviewer points out to us here the parallels with work on the social model of disability. We propose to explore the parallels in a future article. For an overview and retrospective on thirty years of the social model, by its chief architect, see Oliver, M. (2013). “The social model of disability: Thirty years on”. *Disability & Society, 28*, 7.

22
Fowler, N. (2014). *AIDS: Don't die of prejudice*. London: Biteback Publishing.

23
There is, of course, much literature which discusses metaphor and HIV/AIDS. See, for example, Crimp, D. (2004). *Melancholia and moralism: Essays on AIDS and queer politics* Cambridge MA: MIT Press; Garcia‐Duttman, A. (1996). *At Odds with AIDS: Thinking about a virus* Stanford University Press; Haver, W. (1997). *The body of this death: Hitoricity and sociality in the time of AIDS* Stanford University Press; Sontag, S. (1989). *AIDS and its metaphors* London: Penguin; Treichler, P. (1999). *How to have theory in an epidemic: Cultural chronicles of AIDS* Duke University Press; Watney, S. (1994). *Practices of freedom: Selected writings on HIV/AIDS* Duke University Press; Yingling, T. (1997). *AIDS and the national body*. Duke University Press. This said, as we remark in the notes 19 & 20, we are here concerned not with specific metaphors, but rather to make a case for the way in which conceptual metaphors work as frames via which certain constraints (and on occasions also enabling conditions, via a liberating metaphor) are operational sub‐propositionaly. We are putting forward an argument for *why* metaphors matter in understanding shame, the way in which conceptual metaphors can form part of the conceptual anatomy of shame and stigma. We thank an anonymous reviewer for directing us to some of the texts we cite in this note.

24
See Hutchinson, P., & Read, R. (2014). Reframing health care: Philosophy for medicine and human flourishing. In M. Loughlin (Ed.) *Debates in Values‐Based Practice: Arguments for and Against* (pp. 69–85). Cambridge: Cambridge University Press. Fulford, K. W. M. (2014). Values‐based practice: The facts. In M. Loughlin (Ed.) *Debates in values‐based practice: Arguments for and against* (pp. 3–20). Cambridge: Cambridge University Press.

25
British HIV Association Standards of Care for People Living with HIV 2013. Retrieved from http://www.bhiva.org/standards-of-care-2013.aspx/documents/Standards-of-care/BHIVAStandardsA4.pdf

